# Equipping Change Agents: Applying Mixed Methods to Learn About the Outcomes of the Co-Designed Caregiver-Centered Care Champions Education Program

**DOI:** 10.3390/ijerph22101593

**Published:** 2025-10-20

**Authors:** Tanya L’Heureux, Jasneet Parmar, David Nicholas, Lesley Charles, Cheryl Pollard, Myles Leslie, Kimberly Shapkin, Shannon Saunders, Cindy Sim, Paige Walker, Ginger Bitzer, Safia Khalfan, Sharon Anderson

**Affiliations:** 1 Department of Family Medicine, Faculty of Medicine & Dentistry, University of Alberta, Edmonton, AB T6G 2T4, Canadajasneet.parmar@albertahealthservices.ca (J.P.); lesley.charles@albertahealthservices.ca (L.C.); bitzer@ualberta.ca (G.B.); 2 Faculty of Social Work, University of Calgary, Calgary, AB T2N 1N4, Canada; nicholas@ucalgary.ca; 3 Faculty of Nursing, University of Regina, Regina, SK S4S 0A2, Canada; cheryl.pollard@uregina.ca; 4 Department of Community Health Sciences, Cumming School of Medicine, University of Calgary, Calgary, AB T2N 2T8, Canada; myles.leslie@ucalgary.ca; 5 Faculty of Nursing, University of Calgary, Calgary, AB T2N 4L6, Canada; kimberly.shapkin@ucalgary.ca; 6 Assisted Living Alberta, Edmonton, AB T5J 3E4, Canada; shannon.saunders2@albertahealthservices.ca; 7 Team CarePal, Edmonton, AB T5J 1W8, Canada; cindy@teamcarepal.com; 8 Alberta Council on Aging, Edmonton, AB T5M 2T9, Canada; pmwalker16@gmail.com; 9 Independent Consultant, Calgary, AB T3L 0G8, Canada; safiakhalfan82@gmail.com

**Keywords:** family caregivers, caregiver-centred care, co-design, health workforce education, change management, Kirkpatrick-Barr framework

## Abstract

Family caregivers provide most daily care for people living with chronic illness or frailty, yet they remain under-recognized in health and social care systems. To address this gap, we co-designed the Caregiver-Centered Care Champions Education Program, which equips frontline providers with the competencies needed to lead caregiver-inclusive change. Guided by the Kirkpatrick-Barr Health Workforce Education Framework, we conducted a mixed methods interpretive description evaluation of learner satisfaction, knowledge and confidence gains, and self-reported behaviour change. Sixty-seven interdisciplinary participants completed three online modules. Quantitative results from pre/post surveys (Wilcoxon signed rank tests) showed significant improvements across all competencies (*p* < 0.001; large effect sizes) alongside high satisfaction (means 6.56–6.96/7). Qualitative findings revealed that 94% of participants applied program content within three months, and 61% implemented five or more distinct behaviour changes (e.g., collaborative care planning, system navigation support). The analysis illuminated how learners integrated caregiver-centred principles with change leadership strategies. Time constraints and staffing shortages emerged as key barriers. Our co-designed, theory-informed approach effectively bridged individual learning and system change, demonstrating the potential to transform caregiver inclusion practices when supported by organizational policies.

## 1. Introduction

Over the past two decades, health systems have widely adopted patient-centered care, which has improved diagnosis, treatment speed, and pathway coordination [1,2]. Yet family caregivers, who are the largest care workforce, remain largely invisible in health, social, and community care systems [3,4].

We define family caregivers (also called care partners or informal carers) as unpaid relatives, friends, neighbours, or others who provide practical, emotional, advocacy, and coordination support to a person of any age experiencing physical or mental health conditions, disability (including developmental or cognitive disability), substance-use or neurodevelopmental needs, injury, or age-related needs. “Family” includes both families of origin and families of choice, consistent with culturally diverse definitions of kin and support networks. This definition excludes paid staff, unless we are referring to double-duty caregivers (paid providers who also provide unpaid care at home) [5,6].

In Canada, approximately one in four adults—roughly 7.8 million people—take on this unpaid role [7,8]. Family caregivers provide between 75 and 90 percent of daily care for people living in community homes [9,10,11]. Even after a person moves into assisted living or long-term care, family caregivers continue to provide between 15 and 40 percent of direct care [12,13]. The conservative estimated value of these contributions exceeds 97 billion dollars annually [7,8]. However, escalating care demands, combined with limited preparation and recognition, have led to rising levels of caregiver distress [14].

National data show that both the length and intensity of caregiving have increased. For example, the proportion of caregivers providing ten or more hours of care per week rose from 26 percent in 2012 to 35 percent in 2018, while the proportion who reported that they had a choice about taking on caregiving fell from 57 percent to 42 percent [14]. Several factors explain these changes. Smaller family networks and higher participation of women in the workforce reduce the number of available unpaid caregivers. Hospital avoidance and early discharge policies shift complex care responsibilities into the home. Longer survival with chronic illness or disability extends caregiving duration. Family caregivers increasingly perform tasks once carried out by professionals, such as medication management, wound care, symptom management, and system navigation and advocacy. These demands are consistently associated with caregiver psychological distress, sleep disruption, financial strain, and reduced labour-force participation [15,16,17,18,19,20].

Unmet caregiver needs are not only personal hardships but also system-level risks. High caregiver strain is associated with increased emergency department visits, hospital readmissions, and earlier transitions to residential care for the person receiving care [7,8,9,10,11,12]. For caregivers themselves, the consequences include heightened risks of depression, injury, and income loss, with ripple effects across families and the wider economy [21,22,23]. Despite this, care plans rarely document caregivers’ contributions, seek their insights, or assess their capacity to continue caregiving [21,22]. A persistent assumption remains that “someone in the family will manage the plan.” As needs evolve and new providers join the care team, responsibilities are often shifted informally to caregivers—typically without documentation or assessment of their capacity and workload. This results in gaps between prescribed treatment and home-delivered care, and it widens the disconnect between professional intent and unpaid care realities [23,24,25,26,27,28,29].

Although many frontline providers recognize caregiver strain, they face pressures that limit their ability to respond, including time constraints, staffing shortages, and misaligned incentives. Because caregiver support is rarely included in key performance indicators or reimbursement, task-shifting occurs without systematic assessment or documentation. This creates unmet needs for families and moral distress for clinicians [24,30,31,32,33].

To address these barriers, we previously co-designed and evaluated a competency-based Caregiver-Centered Care Education program, consisting of Foundational and Advanced modules (about seven hours of open access learning) [34,35,36]. These modules equip learners to: (1) recognize family caregivers as partners on the care team, (2) collaborate in assessment and care planning, (3) identify and respond to caregiver needs, risks, and preferences, and (4) connect families to health and community supports. The education emphasizes practical competencies and day-to-day behaviours, such as communication, identification, documentation, and referral.

The Caregiver-Centered Care Champions Education program was developed as a subsequent step by the same co-design team. The earlier Foundational and Advanced education focuses on building caregiver-centered competencies. The Champions curriculum adds the dimensions of change leadership and implementation capability. It equips learners with strategies for embedding and spreading caregiver-centered care within teams and organizations, including structured approaches to change management such as Kotter’s model [37] the Prosci ADKAR Model (Awareness, Desire, Knowledge, Ability, and Reinforcement) [38,39], and Plan Do Study Act [PDSA] cycles [40,41,42], which are widely used in quality improvement.

Health systems often rely on champions to spread evidence-based practices [43,44,45,46]. However, reviews show that most champion models depend on a single site-nominated individual, provide brief and information-heavy training, and have limited theoretical grounding or mentorship. Effectiveness often depends on individual motivation rather than systematic preparation [47,48]. We sought to address these gaps by developing the Caregiver-Centered Care Champions Education, a competency-based, evidence-informed, and theoretically grounded program aligned with best practices in healthcare workforce education [38,49,50,51]. Our goal was to prepare learners to reshape mindsets, practices, and organizational culture by equipping them to lead caregiver-inclusive change.

This paper outlines the design of the Champions Education and evaluates its impact using the first three levels of the Kirkpatrick-Barr Health Workforce Education Framework: learner reaction, knowledge and attitude change, and self-reported behaviour [52,53].

### 1.1. Caregiver-Centred Care Champions Education

The Caregiver-Centered Care Champions Education program trains both frontline staff and family caregivers to lead measurable improvements in the recognition, inclusion, and support of family caregivers across hospitals, primary care, community agencies, and long-term care. Champions model caregiver-centered practice, collaborate with families on care planning, share knowledge through mentoring and in-service teaching, and lead structured change initiatives to embed and sustain these practices.

#### 1.1.1. Alignment with Best-Practice Education Design

The curriculum reflects best practices in healthcare workforce education. It was designed using modular micro-learning, authentic scenarios, explicit learning objectives, opportunities for reflection and feedback, and options for asynchronous and facilitated delivery. These design features were selected to maximize accessibility, relevance, and transfer of learning into practice [49,50,51].

#### 1.1.2. Mechanism for System Change

The program positions Champions as boundary-spanning change agents. Champions apply a train-the-trainer model, use small tests of change through Plan Do Study Act (PDSA) cycles in team huddles and rounds, and implement workflow tools such as caregiver identification and assessment prompts, documentation cues, and electronic medical record flags [40,41,42]. These strategies “hard-wire” caregiver-centered practices at the local level. Spread across organizations is supported by a community of practice and by alignment with leadership priorities, which help to sustain momentum and normalize new behaviours.

#### 1.1.3. Participant Recruitment and Enrolment

We recruited participants between August 1 and September 15, 2023, through email invitations, co-design networks, and targeted social media. Eligible learners included experienced family caregivers, frontline health and social service providers, educators, and leaders across care settings. We specifically targeted individuals who had completed the foundational and advanced education and demonstrated motivation, confidence in their ability to influence others, digital fluency, and local leadership potential.

#### 1.1.4. Recognition and Credentialing

Graduates of the program receive a Continuing Competency Certificate in Caregiver-Centered Care. Accreditation with professional colleges is in progress. The learning outcomes are aligned with the six domains of the Caregiver-Centered Care Competency Framework Caregiver-Centred Care Competency Framework [36] and stretched beyond based on appreciative inquiry [54,55], quality improvement [56,57], and change management [37,38].

### 1.2. Theoretical and Evidence Base

#### 1.2.1. Co-Design as a Foundational Philosophy

We grounded the program in the principle that those who deliver, receive, and rely on care should have a voice in shaping the systems that support them [58,59,60,61]. Family caregivers, providers, educators, and leaders all participated as co-designers and ultimately as Champions. A 146-member co-design team collaborated to create the curriculum, bringing together what we describe as “voices of intent, capability, experience, and design” [62]. This approach ensured both academic rigor and practical relevance.

Between August 2022 and September 2023, we held six virtual co-design meetings. Breakout discussions were recorded and transcribed to capture detail. From September to December 2023, fifty participants met virtually three additional times to develop and test program content and tools. Opportunities for input were also offered through email and one-to-one conversations, which allowed for flexible participation.

#### 1.2.2. Rights- and Values-Based Orientation

The program was designed within a rights- and values-based framework, rooted in the Canadian Charter of Rights and Freedoms [63] and principles of person-centered care ethics [64,65]. We operationalized five values through concrete learning tasks:

- Respect: documenting caregiver preferences.
- Meaningful participation: involving caregivers as co-facilitators and case examples.
- Equity: ensuring diverse scenarios, plain language, and asynchronous access.
- Organizational accountability: embedding caregiver recognition through electronic medical record prompts and audit-and-feedback tools.
- Empowerment: encouraging learners to set specific, measurable, attainable, relevant, and time-bound (SMART) goals [66,67], use Plan Do Study Act (PDSA) cycles [40,41,42], and participate in peer coaching.

Pedagogically, we applied sociocultural and transformative adult learning principles as well as constructivist design [68,69,70,71]. Instructional methods included caregiver video vignettes, guided reflection and feedback, and authentic workplace problems that led to usable tools or practices. Appreciative inquiry methods encouraged learners to identify and build on local strengths [54,55].

### 1.3. Curriculum and Learning Process

#### 1.3.1. Structure of the Learning Modules

The program consisted of three asynchronous online modules, each lasting 45 to 60 min, with optional live facilitated discussions to enhance reflection and peer exchange. The modules were:

- Module 1: Inspiring Change—focused on leadership self-awareness and creating a compelling caregiver-centered vision.
- Module 2: Leading Change—focused on stakeholder mapping, building team capacity, and transforming resistance into collaboration.
- Module 3: Managing Change—focused on adaptation cycles, outcome metrics, and sustainability planning.

#### 1.3.2. Instructional Strategies

We used a variety of instructional strategies to maximize engagement and transfer into practice. These included caregiver video vignettes, empathy mapping, role-play, and guided reflection exercises. Learners were also provided with downloadable tools to support practice change in their own settings.

#### 1.3.3. Outputs and Practical Application

By the end of the program, participants produced a concrete change management action plan that they could apply in their workplace. This included a vision statement, a baseline assessment of current practice, a chosen micro-practice for improvement, a process measure, and a sustainment plan.

#### 1.3.4. Community of Practice

After completing the modules, participants were invited to join a community of practice. These groups provided opportunities for peer mentorship, problem-solving, and sharing of strategies to address barriers. They also created a forum for sustaining motivation and extending learning beyond the initial course.

The curriculum was organized into three modules: Inspiring Change, Leading Change, and Managing Change. Together, these modules guided learners through a six-stage cycle: Dream, Discover, Determine, Design, Develop, and Drive Forward.

#### 1.3.5. Learners Produced Practical Outputs at Each Stage, Including:

- a one-sentence vision and “why now” statement,
- a baseline scan and stakeholder map,
- the selection of one micro-practice (for example, identifying caregivers at the first point of contact),
- a simple tool (such as an electronic medical record prompt or pocket card),
- a one-shift Plan Do Study Act (PDSA) cycle [40,41,42], and
- a sustainment plan using the Prosci ADKAR Model (Awareness, Desire, Knowledge, Ability, and Reinforcement) [38,39].

This six-stage cycle was reinforced by leadership strategies adapted from Kotter [37], Prosci’s Model for Improvement [38] with its Plan Do Study Act cycles [40,41,42]. Learners also had ongoing access to an online resource hub to support implementation and sustainment.

### 1.4. Intended Contribution

#### 1.4.1. Integration of Theory and Practice

The Champions Education program was designed to integrate theory, co-designed curriculum, and an implementation “practice bundle.” This combination enables frontline staff and leaders to embed small but meaningful changes that make caregiver partnership visible and routine.

#### 1.4.2. The Practice Bundle

The bundle included practical templates and scripts for caregiver identification and assessment, shared decision-making, and teach-back communication. It also included sample wording for electronic medical record documentation and a set of quality improvement tools, such as stakeholder maps, driver diagrams, SMART goal planners, and Plan Do Study Act (PDSA) cycle run charts [40,41,42].

#### 1.4.3. Adaptation to Local Contexts

These tools were designed to be adapted and tested in local workflows. Learners used them to run small-scale tests of change and to refine practices based on real-world conditions. This approach encouraged ownership, contextual fit, and sustainability.

#### 1.4.4. Program Summary and Supplementary Materials

A concise summary of the program was provided earlier in this paper, and full operational details are included in Supplementary Materials S1.

## 2. Materials and Methods

### 2.1. Study Design and Theoretical Framing

We used a mixed methods interpretive description design to evaluate the program. Interpretive description is an approach well suited to applied health disciplines because it generates insights that directly inform practice [72,73,74].

The Kirkpatrick-Barr Health Workforce Education Framework [72,73,74] guided our evaluation. We assessed outcomes across three levels:

1. Learner satisfaction and engagement,
2. Changes in knowledge, skills, and attitudes, and
3. Self-reported behaviour change.

We obtained ethical approval from the University of Alberta Research Ethics Board (Pro00138770; Pro00109366). We also followed the Good Reporting of a Mixed Methods Study (GREET) reporting guidelines [75].

### 2.2. Participant Recruitment and Educational Context

We recruited participants between 1 August and 15 September 2023. Recruitment was conducted through invitations to individuals who had completed the Foundational and Advanced caregiver-centered care education, through outreach to members of the co-design team, and through targeted social media posts.

To be eligible, participants had to:

- Be a health, social, or community care provider or leader, or an experienced family caregiver,
- Have completed the Foundational and Advanced modules (or committed to completing them before the first session), and
- Have access to an internet-enabled device.

Participation was voluntary, and all participants provided electronic consent.

A total of 67 individuals enrolled. Of these, 62 participants (92.5 percent) completed the full program, and 49 completed the three-month follow-up survey.

The program curriculum consisted of three asynchronous online modules (each 45–60 min) with optional live discussions. Learners produced a vision statement, a baseline scan and stakeholder map, the selection of one micro-practice for improvement, a local tool such as an electronic medical record prompt, a one-shift Plan Do Study Act (PDSA) cycle with a process measure, and a sustainment plan. Additional details are provided in Supplementary Materials S1.

### 2.3. Data Collection Instruments

We developed all evaluation instruments using a theory-driven approach. Each item was mapped to the Caregiver-Centered Care Competency Framework [36], to change leadership theory (Kotter; ADKAR) [37,38], and to contemporary education evaluation principles [76]. Our design was also informed by rights- and values-based adult learning orientations [68,70,77].

Survey stems were adapted from validated items used in previous evaluations of caregiver-centered care education [35,78,79]. We refined the instruments through co-design input and pilot testing. Surveys were administered electronically through Google Forms, and data were exported to Excel.

- Level 1 (Reaction and Satisfaction, immediate post-course): Five Likert-scale items and one open-ended question captured learner engagement, perceived relevance, perceived usefulness, strengths, areas for improvement, and likelihood of recommending the course.
- Level 2 (Knowledge, Skills, and Confidence, pre- and post-course): A nine-item Caregiver Champions Knowledge and Confidence Scale (7-point Likert; Cronbach’s α = 0.82–0.91). Pre and post surveys were linked anonymously by participant-chosen codes.
- Level 3 (Behaviour Change, three-month follow-up): The same scale was re-administered, along with additional items assessing enactment of caregiver-centered practices, progress on SMART goals, and perceived barriers and enablers.

Full versions of the instruments are provided in Supplementary Materials S2.

### 2.4. Data Analysis

Quantitative analysis: We summarized demographics and satisfaction using descriptive statistics. Paired-sample *t*-tests were used to assess pre- and post-program changes, with Cohen’s *d* used to estimate effect sizes.

Qualitative analysis: We conducted inductive content analysis [80,81] of open-ended survey responses at all three Kirkpatrick-Barr levels. Two researchers independently reviewed the data, coded transcripts using NVivo, and grouped codes into categories. We then refined these into themes aligned with the program modules and Kirkpatrick-Barr levels. We enhanced credibility through dual coding, iterative discussions, and interpretive consensus.

Finally, we integrated the qualitative findings with the quantitative results to provide a fuller understanding of how learners applied caregiver-centered care principles and leadership strategies in practice.

## 3. Results

### 3.1. Participant Characteristics

We recruited 67 participants from diverse professional backgrounds: nurses (n = 29, 43%), social workers (n = 7, 10%), recreation therapists (n = 6, 9%), occupational therapists (n = 5, 7%), caregivers (n = 5, 7%), physiotherapists (n = 3, 4%), dietitians (n = 2, 3%), and others (e.g., youth worker, psychologist, SLP, primary care assistant, healthcare aide, physiotherapy assistant, kinesiologist, not-for-profit leader (n = 2), business owner; each 1–3%). Settings included home care (n = 18, 27%), acute care (n = 16, 24%), community services (n = 15, 22%), long-term care (n = 10, 15%), primary care (n = 6, 9%), and palliative care (n = 2, 3%).

The majority of participants identified as female, with only six participants (9%) identifying as male. Most were mid-career professionals: 26 were aged 45–54 (39%), and 23 were aged 35–44 (34%). Younger participants included eight aged 26–34 (12%) and one aged 21–25 (1%), while older participants included six aged 55–64 (9%) and three aged 65 or older (4%). No participants were aged 20 or younger. Completion rate was 92.5% (62/67); 49 (73.1%) completed the 3-month survey.

### 3.2. Results of the Caregiver-Centered Care Champions Education

Participants reported high satisfaction, meaningful knowledge gains, and substantial changes in attitudes, behaviours, and clinical practice. Qualitative findings across survey and interview data also demonstrated how participants were applying the core competencies in their day-to-day work and internalizing the leadership lessons embedded in the Champions modules.

#### 3.2.1. High Satisfaction and Relevance (Kirkpatrick-Barr Level 1)

Mean satisfaction ratings ranged 6.56–6.96/7 (median/mode = 7). Learners valued clarity, relevance, and asynchronous delivery. Participants described the course as energizing and validating, with one stating, “*It has reinvigorated my energy and increased my skill set in making change.”* Another reflected, *“It was great to connect with others who have a vested interest in this type of work… It made me feel less alone or isolated in this journey*”.

Participants who attended the facilitated learning sessions valued hearing from others across sectors, which helped them reflect on their own practice and envision systemic change: “*I have loved learning from others around shared visions and different perspectives and learning needs—seeing how they are looking to make practice changes*”.

#### 3.2.2. Significant Increases in Knowledge and Confidence (Kirkpatrick-Barr Level 2)

Distribution checks indicated non-normality; Wilcoxon tests showed significant increases in total Knowledge and Confidence scores (Z = −6.21, *p* < 0.001; r = 0.80). All items improved (*p* ≤ 0.001; r = 0.43–0.71), with the largest shifts for implementation planning and overcoming barriers. See Table 1 Wilcoxon Rank Tests Pre-Post Education Parametric corroboration. Paired *t*-tests showed the same pattern (all t(60) |−3.8|–|7.5|, *p* < 0.001), with large Cohen’s *d* across items (0.65–0.96; largest for question 9 d = 0.96; smallest for question 5 d = 0.65). See Table S1 Paired Samples *t*-tests with Cohen’s effect sizes.

In the qualitative data, the strongest reported improvement related to reflective practice:

*“It reminded me to listen closely and ensure I paraphrased the need. It helped me recognize strengths and encouraged me to stretch my thinking about what I or others can do.”*

Others noted the practical ways the course enhanced their confidence in working within teams:

*“I work in the community setting, so the course gave more concrete examples of how different healthcare professionals could realistically be actively supporting caregivers.”*

Several participants began to recognize how small shifts in communication could translate into big differences in caregiver trust and engagement. As one noted, “*I now ask caregivers what they need, rather than assuming*”. Three participants reported they had been caregiver champions for years, so while they did not think it added to their knowledge and confidence, welcomed the course to develop more champions, “*Not a lot of new learning with my 30 years of experience, but I welcome the course for the opportunity for new colleagues to carry the torch*”.

#### 3.2.3. Behavioural Change and Application in Practice (Kirkpatrick-Barr Level 3)

Three to six months after completing the Caregiver-Centered Care (CCC) Champions Education Program, 46 of 49 (93.8%) respondents reported making changes in their caregiving-related behaviours and clinical practices. While 38.7% reported making between one and four changes, the majority—61.3%—had implemented five to eight distinct changes. This suggests that the program had a strong impact not just on learners’ knowledge and attitudes, but on their day-to-day practice.

**Figure 1:**
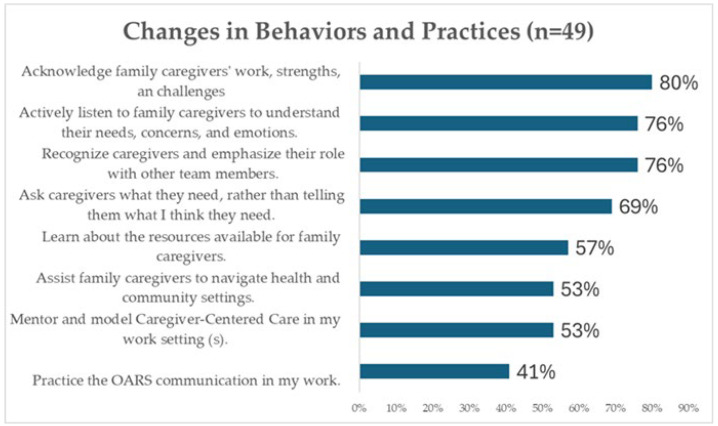
Changes in Behaviours and Practices.

These self-reported behaviour changes strongly aligned with the four Caregiver-Centered Care Core Competencies:

- 80% began to more intentionally acknowledge caregivers’ contributions and challenges.
- 76% recognized caregivers as essential members of the care team and emphasized this with colleagues.
- 76% actively listened to caregivers to better understand their needs and emotions.
- 69% moved from prescriptive approaches to more collaborative care planning—asking caregivers what support they required rather than assuming. See Figure 1 for Changes in Behaviors and Practices.

These quantitative results were further supported by qualitative reflections from learners, which illustrated how the CCC core competencies and Champions Education modules were integrated into practice. Learners described how the program reinforced caregiver-inclusive values, inspired team discussions, and catalyzed new roles or programs. For example:

“I became more like a listener who listens to families’ needs and their expectations.”

“I now step back and ask what the caregiver thinks they may need instead of overwhelming them with information.”

“I created a family/caregiver resource document and check in with them to ensure they know we care about them and are here to support them.”

These changes reflect participants’ deepened commitment to the core CCC competencies:

- Recognizing caregivers as team members
- Partnering in care planning
- Assessing and supporting caregiver needs
- Guiding caregivers through health and community systems

Although the Champions Education primarily focused on change leadership and system improvement, many learners reported re-engaging with foundational caregiver-centered principles through the program’s reflective and action-oriented components. This included structured planning activities, Plan Do Study Act (PDSA) cycles, and personal vision development, which helped participants internalize and reapply the core competencies in practical ways.

The integration of both CCC content and change leadership skills led participants not only to implement changes themselves, but to influence their teams and work settings. These included new documentation practices, peer education efforts, and caregiver-inclusive initiatives. Table 2 below summarizes key themes and exemplar quotes reflecting both CCC competencies and the three modules of the Champions Education.

#### 3.2.4. Enablers and Barriers to Implementation

Enablers: time to interact with caregivers (78.6%), positive relationships (76.2%), colleague support (50.0%), leadership support (40.5%).

Barriers: lack of time (51.2%), competing demands (46.5%), staffing shortages (41.9%), leadership resistance (16.3%).

Time was both enabler and barrier. Team/leadership support influenced embedding practices in workflows; systemic constraints underscored the need for policy and workflow alignment.

## 4. Discussion

Our mixed methods evaluation indicates that the Caregiver-Centered Care Champions Education program produced meaningful gains in knowledge, confidence, and self-reported behaviour related to caregiver engagement and leading change. Although the Caregiver-Centered Care Competency Framework [36] underpinned the program, the instructional focus extended beyond competency acquisition to emphasize change leadership and implementation. Large and consistent pre-post improvements suggest that participants developed stronger perceived capacity to lead change, which conceptually aligns with constructs in the Theory of Planned Behaviour [80,81].

### 4.1. Positioning Relative to Prior Programs

These findings build on earlier evaluations of the Caregiver-Centered Care Education Foundational and Advanced modules, which emphasized awareness and practice skills [34,35]. They also align with broader workforce education programs where features such as modular design, reflective practice, and authentic scenarios support knowledge transfer [49,50,51]. The Champions curriculum extends this literature by integrating change leadership and quality improvement approaches, including Kotter’s model, the Prosci ADKAR Model (Awareness, Desire, Knowledge, Ability, and Reinforcement), and Plan Do Study Act (PDSA) cycles [37,38,39,40,41,42]. In addition, the inclusion of experienced family caregivers as co-learners addressed limitations of traditional “champion” programs, which often rely on brief, information-heavy training with little theoretical grounding or mentorship [47].

### 4.2. Why Leadership-Oriented Approaches Matter Now

Family caregivers deliver the majority of daily care, between 75 and 90 percent in community settings [9,10,11], yet their roles are often overlooked in health, social, and community care planning and documentation [82,83,84]. As health systems shift more complexity into the home, responsibilities are transferred to caregivers without assessment of their capacity [29,85,86], which contributes to caregiver distress and system inefficiencies [16,17]. At the same time, in our patient-focused systems, clinicians face time pressures, staffing shortages, and incentives that are not aligned with caregiver support [29,87].

Preparing staff to lead practical, unit-level change is one promising way to narrow the gap between system intent and caregiver experience. In our program, learners created unit-specific tools, facilitated short in-service sessions, and applied Plan Do Study Act (PDSA) cycles to test practices such as caregiver identification at the first point of contact. These activities cultivated shared ownership, flattened hierarchies, and engaged colleagues in collaborative practice. Such mechanisms are consistent with literature on co-design and knowledge mobilization [29,85,88,89].

### 4.3. Barriers, Transfer Climate, and Sustainment

Our evaluation highlighted enduring challenges. Participants reported that lack of time, competing priorities, and staffing shortages constrained their ability to sustain new practices. These factors reflect the transfer climate, which strongly shapes whether learning is adopted and maintained. Research suggests that lasting change requires visible leadership endorsement, shared team norms, protected time, and straightforward ways to measure and communicate progress [40,41,45,56,88,89].

The Champions model addressed some of these conditions by combining train-the-trainer approaches, small tests of change within team huddles, workflow reminders such as electronic prompts, and peer-to-peer support through communities of practice. Together with leadership alignment, these elements appear to increase feasibility in complex settings [40,41,45,56,88,89].

### 4.4. Implications for Systems Change

Our findings point to practical steps that organizations can take to embed caregiver-centered care at scale. These include:

- Integrating caregiver identification and assessment templates into workflows, including electronic medical records.
- Normalizing collaborative care planning as standard practice [90,91].
- Using brief communication techniques such as teach-back [92] and goal-setting [66] within routine encounters.
- Resourcing communities of practice so that Champions can mentor peers and address barriers as they arise [93,94].

### 4.5. Strengths and Limitations

Strengths of this study include a theory-informed design guided by the Kirkpatrick-Barr Health Workforce Education Framework, co-design with a large and diverse stakeholder group, the use of mixed methods, and high program completion rates. Because learners in this cohort had previously completed the Foundational and Advanced Caregiver-Centered Care Education, they may have had a positivity bias toward caregiver-inclusive practice. Limitations include reliance on self-reported outcomes, a modest sample size, and contextual specificity that may limit generalizability. Participants were predominantly health and social care professionals, with a smaller subset of experienced family caregivers. We did not directly measure outcomes for family caregivers receiving support; findings reflect learner perceptions and self-reports at follow-up. Attendance at optional live sessions was not systematically tracked.

### 4.6. Recommendations

Based on our findings, we recommend the following actions:

1. Scale and spread the program: Extend the Caregiver-Centered Care Champions Education to additional care settings and regions, prioritizing acute care, emergency departments, and rehabilitation services where caregiver visibility remains low [21,22,23].
2. Strengthen leadership engagement: Embed expectations for caregiver-centered practice in policy frameworks, job descriptions, performance metrics, and accreditation standards [95].
3. Support communities of practice: Provide ongoing opportunities for Champions to sustain momentum, share strategies, and access peer mentorship through structured follow-up or regional learning hubs [21,22,23].
4. Reinforce behaviours through structures: Create organizational tools and policies that formally recognize caregiver roles, such as caregiver assessment templates and documentation prompts [95].
5. Address implementation barriers: Integrate caregiver-centered care into existing workflows and team models, and advocate for protected time to support caregiver engagement activities [65,66,95].
6. Normalize caregiver education: Include caregiver-centered education in the foundational training of health, social care, and community trainees so that partnership with caregivers becomes standard practice [29,50,57,96].
7. Expand evaluation metrics: Move beyond learner self-report to assess caregiver outcomes, team function, and system-level measures such as documentation reliability in electronic medical records [29,85,87].

### 4.7. Implications for Practice

For interprofessional teams, caregiver partnership can be advanced by identifying and documenting the caregiver at first contact, inviting caregivers into care planning and bedside handovers, and applying communication techniques such as teach-back and goal-setting during routine workflows. Unit-based Champions can coach peers and run Plan Do Study Act (PDSA) cycles to reinforce these practices [40,41,49,50,51,56,95,97]. Electronic prompts in medical records and structured team huddles can operationalize warm handovers to community supports and strengthen role clarity [98,99]. Collectively, these actions make caregiver partnership feasible in time-pressured environments while improving continuity and safety.

### 4.8. Future Research

Future research should use feasibility and implementation frameworks such as Reach, Effectiveness, Adoption, Implementation, and Maintenance (RE-AIM) [100,101] and the Consolidated Framework for Implementation Research (CFIR) [102,103] to assess program spread. Priority areas include reach, adoption, fidelity, time, costs, and sustainability. Co-designed outcome measures for caregivers, as well as team and system metrics such as electronic documentation reliability, will be critical to capture the broader impact of caregiver-centered change.

## 5. Conclusions

The Caregiver-Centered Care Champions Education program extends beyond traditional workforce training by positioning family caregivers and professionals as co-learners and by integrating change management and quality improvement methods with a rights- and values-based competency framework. Participants reported increased knowledge, confidence, and leadership capacity, and they described practical changes in communication, care planning, and advocacy.

By coupling education with implementation strategies such as Plan Do Study Act (PDSA) cycles, stakeholder mapping, and workflow prompts, the program enabled participants to embed caregiver-centered practices in their own contexts. Early outcomes suggest that this model can bridge the gap between individual learning and system change when supported by leadership and organizational policies.

Future work should test the program on a larger scale and evaluate its impact on caregivers, care recipients, and system performance using co-designed outcome measures. Preparing teams to integrate family caregivers as partners in care is both feasible and essential to advancing quality, safety, and sustainability in health and social care.

## Figures and Tables

**Table 1:** Wilcoxon Signed Rank Test.

	Z	Asymp. Sig. (2-Tailed)
Pre and Post Questions.	−6.21b	0.000
I actively reflect upon existing strengths and opportunities to bring Caregiver-Centered Care to my team, organization or network.	−4.40b	0.000
I am aware of what most qualities support me in being a Caregiver-Centered Care Champion.	−4.69b	0.000
I am confident in my ability to inspire others to embrace and spread Caregiver-Centered Care.	−4.60b	0.000
I am confident that I can navigate others resistance to embracing Caregiver-Centered Care.	−5.01b	0.000
I understand how change management and quality improvement principles can guide my work in enhancing Caregiver-Centered Care.	−4.44b	0.000
I know how to create a comprehensive plan to guide the implementation and sustainability of my efforts.	−5.46b	0.000
I actively engage in partnership building to support Caregiver-Centered Care.	−4.58b	0.000
I am committed to building others’ competence to practice Caregiver-Centered Care.	−3.36b	0.001
I am confident that I can overcome barriers to implementing Caregiver-Centered Care.	−5.55b	0.000

b. Based on negative ranks.

**Table 2:** Exemplar quotes mapped to competencies and modules.

Theme	Quote
Recognizing caregivers as team members	*“Encouraging co-workers to find empathy for caregivers. To look beyond what is presented from the caregiver, to why are they presenting/reacting to our team and healthcare in this manner.”* *“Recognition, respect, collaboration and alliance for a better-quality outcome for patient, family and caregiver.”*
Partnering in care planning	*“Working more on empowering versus creating dependence.”* *“Use the CSNAT—the Carer Support Needs Assessment Tool to understand what caregivers need and foster their resiliency!”*
Supporting caregiver needs	*“I utilize the resources available, ensure connection through communication & collaboration, as well as share the website and poster cards I have.”* *“Our Hospice Society will be using these programs in the orientation of our new staff and our volunteers who work with caregivers.”*
Navigating systems	*“I don’t work within the system. Instead, I use my background in science education and lived expertise to try to help others better navigate the system. I found this course helpful in that it made me realize more fully what we’re up against.”*
Reflective practice	*“I’ve always respected the caregivers I met and worked with. This course gave me reminders of times I helped.”*
Inspiring change (Module 1)	*“Now looking at it from the other side—as I was the caregiver—I understand how exhausting it is and how difficult it is to ask for help.”* *“As a double-duty caregiver, I radically changed my thinking about caregiver-centred care.”*
Leading change (Module 2)	*“I’ve incorporated the Caregiver-Centered Care online course as a requirement for students. I hope it helps learners see the crucial role caregivers provide.”* *“I use the materials included in the education.”*
Managing change (Module 3)	*“I routinely meet with caregivers after assessments to validate needs and goals. It’s now part of my practice.”* *“ I was burned out and my new supervisor recommended I take this Champions Education. Working in home care settings, I meet many caregivers, and they motivate me.”*

## Data Availability

The datasets used and/or analyzed during the current study are available from the corresponding author on reasonable request.

## Supplementary Materials

The following supporting information can be downloaded at: https://www.mdpi.com/article/10.3390/ijerph22101593/s1, Supplementary Materials S1: Detailed operational overview of the Caregiver-Centered Care Champions curriculum components, Supplementary Materials S2: Data Collection Instruments. Supplementary Materials S3: Figure S1 Changes in Knowledge and Confidence Pre-Post Education means, Table S1 Paired Samples *t*-test Pre-Post Champions Education.
