# Supplementary material for: Equipping Change Agents: Applying Mixed Methods to Learn About the Outcomes of the Co-Designed Caregiver-Centered Care Champions Education Program
Source: Int J Environ Res Public Health. 2025 Oct 20;22(10):1593. doi: 10.3390/ijerph22101593 (PMC12563762; doi:10.3390/ijerph22101593)
Supplement: Supplementary file 1 [file ijerph-22-01593-s001.zip › ijerph-3844093-supplementary.pdf]

Supplementary Materials

Supplementary Materials S1: Detailed operational overview of the Caregiver-Centered Care Champions curriculum components

Key Elements of the Caregiver-Centered Care Champions Training

The **Foundational** and **Advanced** Caregiver-Centred Care (CCC) courses build individual knowledge and skills. **Champions training** goes a step further: it prepares health, social, and community-care providers and leaders to drive **organisational** change. Co-designed with frontline practitioners, the programme equips you with the communication, leadership, and change-management skills needed to plan, launch, and sustain CCC initiatives in your workplace and community.

Learning Outcomes

Module 1 - Inspiring Change: Builds leadership self-awareness and articulates a compelling caregiver-centred vision.

- Identifying your personal qualities and strengths as a leader.
- Writing both a vision statement and an elevator pitch that captures the change you wish to achieve.

Module 2 - Leading Change: Equips participants to map stakeholders, build team capacity, and transform resistance into collaboration.

- Identifying the individuals and/or groups that will help to bring about change.
- Developing a plan to build knowledge and practice supports for those in your organization who will be implementing Caregiver-Centered Care.

Module 3 - Managing Change: Guides learners through adaptation cycles, outcome metrics, and sustainability planning.

- Identifying ways that change efforts can be adapted to what's working and what is not working.
- Planning for how the effectiveness of the Caregiver-Centered Care changes will be measured and sustained.

At the end of this education, you will be better prepared to champion organizational change.

What are the goals of Caregiver-Centered Care Champions education?
By taking this education, we know that learners care about effectively partnering with and integrating family caregivers into their professional practice. Thank you for being part of this movement.

The goal of these modules is to give learners the tools and thought processes that will help them manage a significant Caregiver-Centered Care initiative in your organization.

Module	Thought Processes	Skills
Module 1:<br>**Inspiring Change**	**Dream:** What could be?	1, Visioning<br>2, Connecting
	**Discover:** What do I do well?	1, Personal leadership<br>2, Inspiring others
Module 2:<br>**Leading Change**	**Determine:** With whom am I leading?	1, Partnerships<br>2, Learning from resistance
	**Design**: How to lead people through change?	1, Building competence<br>2, Sphere of influence
Module 3:<br>**Managing Change**	**Develop**: How can we strengthen our actions?	1, Solutions<br>2, Learning and adapting
	**Drive Forward**: How will we continue, evolve, and improve?	1, Building community<br>2, Sustaining efforts

Learning Outcomes:

At the end of these ***three*** modules, learners will be better prepared to champion caregiver-centered organizational change by:

1. Identify their personal leadership strengths that can drive caregiver-centred change.
2. Craft a concise vision statement and elevator pitch that clearly articulate the desired change.
3. Map key stakeholders and allies whose influence is needed to advance caregiver-centred care.
4. Design an organisational plan to build caregiver-centred knowledge, skills, and practice supports among colleagues.
5. Adapt change strategies in real time, using feedback to reinforce what works and revise what does not.
6. Develop metrics and a sustainability roadmap to measure, report, and maintain the impact of caregiver-centred care initiatives. It is delivered through a flexible, interactive, evidence-informed online learning package that is immediately applicable in the workplace.

Aspect	What the training includes
**Curriculum Structure**	*Three self-paced online modules (~45–60 min each)*<br>1. **Inspiring Change** – the champion role, why caregiver partnership matters, personal leadership mindset.<br>2. **Leading Change** – communication techniques, advocacy messages, motivating peers, handling resistance.<br>3. **Managing Change** – practical change-management tools (SMART goals, stakeholder mapping, PDSA cycles), sustaining gains and measuring impact.
**Core Competencies Built**	• Recognising & documenting caregivers as partners on the care team and "co-clients."<br>• Active listening & shared decision-making skills.<br>• System navigation: linking families to health, social, and community resources.<br>• Assessing caregiver capacity, distress, and risk with validated tools.<br>• Leadership for culture change—coaching colleagues, spreading innovations.<br>• Advocacy for caregiver-friendly policies inside organisations.

Aspect	What the training includes
**Learning Methods**	• Adult-learning theory, Transformative and Constructivist Learning Theory, change-management principles , and the Caregiver-Centred Care Competency Framework.<br>• Video vignettes of real caregivers and providers.<br>• Interactive exercises (e.g., empathy-mapping, communication role-plays).<br>• Downloadable practice tools and templates for immediate use.<br>• Personal development plans created and refined across modules.
**Blended Support**	• Asynchronous platform at *caregivercare.ca/champions* for flexible access.<br>• Three synchronous Zoom sessions for small-group discussion, problem-solving, and peer feedback.<br>• Ongoing Community-of-Practice (quarterly events) to share wins, barriers, and resources.
**Evaluation & Reflection**	• Pre/post knowledge & confidence quiz (9-item Champions Knowledge Assessment).<br>• Learner-written SMART goals to translate training into workplace change.<br>• Three-month follow-up survey on behaviour and practice changes.
**Foundational Frameworks**	• Caregiver-Centered Care Competency Framework.<br>• Kirkpatrick-Barr evaluation levels (satisfaction → knowledge/attitude → behaviour).<br>• Change-management models adapted for health and community settings.
**Outputs for Learners**	• Certificate of completion (“Caregiver Champion”).<br>• Toolkit of ready-to-use resources (conversation guides, assessment checklists, communication templates).<br>• Access to a multi-disciplinary peer network for mentorship and joint projects.

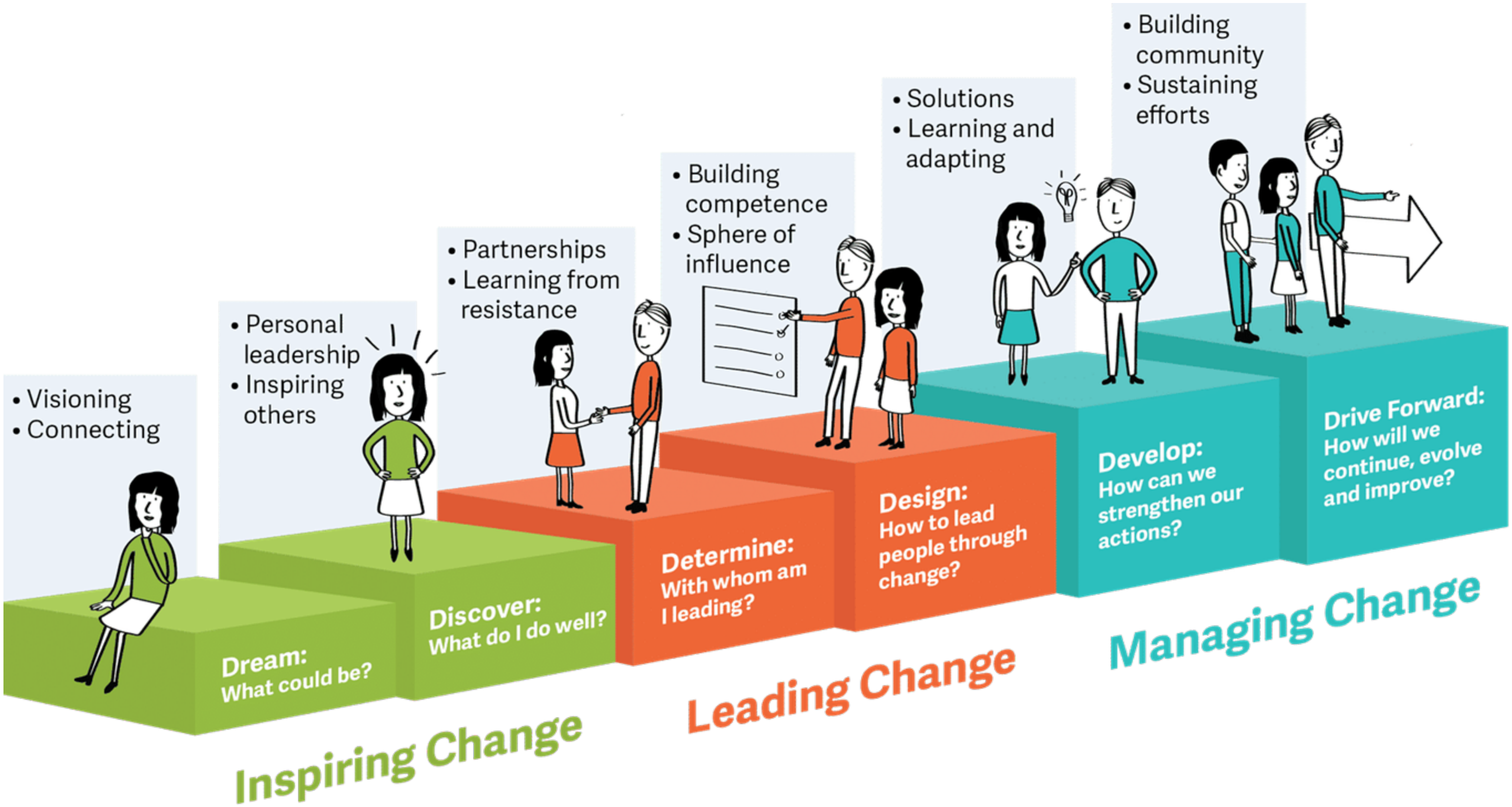

• Visioning
• Connecting
• Personal leadership
• Inspiring others
• Partnerships
• Learning from resistance
• Building competence
• Sphere of influence
• Solutions
• Learning and adapting
• Building community
• Sustaining efforts
Dream: What could be?
Discover: What do I do well?
Determine: With whom am I leading?
Design: How to lead people through change?
Develop: How can we strengthen our actions?
Drive Forward: How will we continue, evolve and improve?
Inspiring Change
Leading Change
Managing Change

Supplementary Materials S2: Data Collection Instruments.

Level 1: Participants Satisfaction with the Education

1. The goals of this education were clear.

2. Overall, the quality of the course content was excellent.

3. The videos helped to increase my understanding of supporting family caregivers.

4. The exercises between the videos increased my knowledge.

5. I am motivated to continuing to learn more about Caregiver-Centered-Care and being a Caregiver-Centered Care Champion.

Level 2: Changes in Participants Knowledge, Comfort and Confidence Before and After the Education

1. I actively reflect upon existing strengths and opportunities to bring Caregiver-Centered Care to my team, organization or network.
2. I am aware of what most qualities support me in being a Caregiver-Centered Care Champion.
3. I am confident in my ability to inspire others to embrace and spread Caregiver-Centered Care.
4. I am confident that I can navigate others resistance to embracing Caregiver-Centered Care.
5. I understand how change management and quality improvement principles can guide my work in enhancing Caregiver-Centered Care.
6. I know how to create a comprehensive plan to guide the implementation and sustainability of my efforts.
7. I actively engage in partnership building to support Caregiver-Centered Care.
8. I am committed to building others' competence to practice Caregiver-Centered Care.
9. I am confident that I can overcome barriers to implementing Caregiver-Centered Care.

Level 3 Survey: Behavior Changes from the Education

1. What Caregiver-Centered Care Education did you complete? Check off all that apply (Foundational, Advanced, Champions)

2. In what health or community care setting do you currently work? or are you a student or trainee? (Answer based on most of your time)

3. What province do you live in?

4. What is your age?

5. Please share your gender (e.g., male, female, transgender, gender neutral, non-binary, agender, pangender, genderqueer, two-spirit, third gender, and all, none or a combination of these).

6. What is your ethnicity?

7. What is your employment role or title? (e.g., team leader, executive director, case manager, business owner, nurse educator)

8. Are you a family caregiver (Carer, care-partner)? We define family caregiver broadly, as family caregiver is any person who takes on a generally unpaid caring role and provides emotional, physical, or practical support in response to physical and/or mental illnesses, disabilities, or age-related needs. Care could be for a family member, chosen family, friend, or neighbor.

Have you considered a change in your employment because of your family caregiving role? It could be working part time, taking a leave of absence, retiring

Can you tell us how you are considering changing your employment because of family caregiving? Or how have you already changed your employment to accommodate your family caregiving?

1. The Caregiver-Centered Care Education gave me new knowledge and skills that have improved my interactions with family caregivers.

2. The Caregiver-Centered Care Education increased my comfort, confidence, and ability to interact with family caregivers.

3. The Caregiver-Centered Care Education improved my communication with family caregivers.

4. Which of the following changes did you make in how you interact with family caregivers?

- Recognize caregivers and emphasize their role with other care team members
- Acknowledge family caregivers work, strengths, and challenges
- Actively listen to family caregivers to understand their needs, concerns, and emotions
- Practice the OARS communication more in my work.
- Ask caregivers what they need, rather than telling them what I think they need.
- Empower the caregiver about the broad spectrum of resources they can take advantage of.
- Learn about the resources available for family caregivers.
- Assist family caregivers to navigate the health and community systems.
- Be the guide who helps family caregivers learn to navigate the health and community systems.
- Mentor and model Caregiver-Centered Care in my work setting (s)
- OTHER changes

5. Facilitators: What helped you to use the Caregiver-Centered Care Education in your practice? Check all that apply. (choice=Leadership support)

- Leadership support
- Support from colleagues
- Time to interact with caregivers
- Positive interactions with family caregivers
- Complements or Thank-you from family caregivers
- Other or I would like to tell you more about the facilitators

6. Barriers: What makes it hard for you to use the Caregiver-Centered Care Education in your practice? Check all that apply. (

- Lack of time
- Heavy workload
- Leadership resistance
- Leadership instability
- Staff Resistance to change
- Staffing Shortages
- Scarce resources
- Competing demands
- Other barriers or I would like to tell you more about the barriers.

7. Can you give us an example of how the Caregiver-Centered Care Education has changed how you work with family caregivers? (Tell us the story of what changed, why, and how the change made you feel).

8. Can you tell us what you need to help to you provide Caregiver-Centered Care to family caregivers in your work setting? It could be anything, policy, a pathway, reimbursement, more time

Supplementary Materials S3: Changes in Knowledge, comfort and confidence before and after the Champions Education

Figure S1 Changes in Knowledge and Confidence Pre-Post Education: Comparison of Pre and Post Education Means

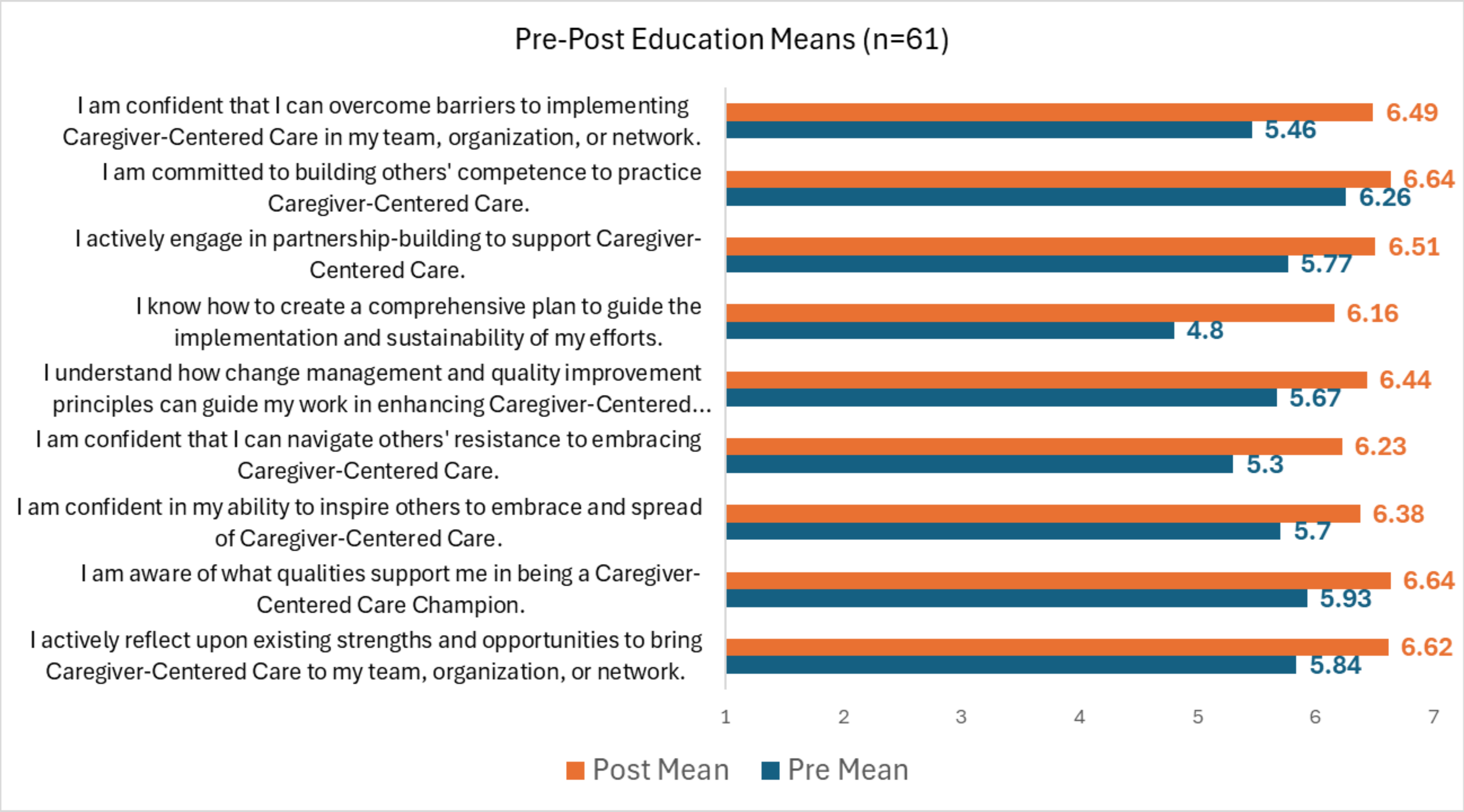

Table S1 Paired Samples t-test Pre-Post Champions Education

	Paired Differences								Cohen's d: Paired Samples Effect Sizes			
Paired Samples Test	Mean	SD	SE Mean	95% Confidence Interval		t	df	Sig	Standardizera	Point Estimate	95% CI	
				Lower	Upper			2-Sided p			Lower	Upper
1. I actively reflect upon existing strengths and opportunities to bring Caregiver-Centered Care to my team, organization, or network	-0.79	1.23	0.16	-1.10	-0.47	-5.01	60.00	<.001	1.23	-0.64	-0.92	-0.36
2 I am aware of what qualities support me in being a Caregiver-Centered Care Champion.	-0.71	0.99	0.13	-0.96	-0.45	-5.57	60.00	<.001	0.99	-0.71	-0.99	-0.43
3. I am confident in my ability to inspire others to embrace and spread of Caregiver-Centered Care.	-0.67	0.85	0.11	-0.89	-0.45	-6.17	60.00	<.001	0.85	-0.79	-1.08	-0.50
4 I am confident that I can navigate others' resistance to embracing Caregiver-Centered Care.	-0.93	1.11	0.14	-1.22	-0.65	-6.58	60.00	<.001	1.11	-0.84	-1.13	-0.55
5 I understand how change management and quality improvement principles can guide my work in enhancing Caregiver-Centered Care in my team, organization, or network.	-0.77	1.19	0.15	-1.08	-0.47	-5.06	60.00	<.001	1.19	-0.65	-0.92	-0.37
6 I know how to create a comprehensive plan to guide the implementation and sustainability of my efforts.	-1.36	1.45	0.19	-1.73	-0.99	-7.33	60.00	<.001	1.45	-0.94	-1.24	-0.63
7 I actively engage in partnership-building to support Caregiver-Centered Care.	-0.74	1.02	0.13	-1.00	-0.48	-5.68	60.00	<.001	1.02	-0.73	-1.01	-0.44
8 I am committed to building others' competence to practice Caregiver-Centered Care.	-0.38	0.78	0.10	-0.58	-0.18	-3.79	60.00	<.001	0.78	-0.49	-0.75	-0.22
9 I am confident that I can overcome barriers to implementing Caregiver-Centered Care in my team, organization, or network.	-1.03	1.08	0.14	-1.31	-0.76	-7.47	60.00	<.001	1.08	-0.96	-1.26	-0.65
Pre- Post Total Scale Score	-7.38	6.25	0.80	-8.98	-5.78	-9.21	60.00	<.001	6.25	-1.18	-1.50	-0.85
